# The combination of IL-2 nanoparticles and Palbociclib enhances the anti-tumor immune response for colon cancer therapy

**DOI:** 10.3389/fimmu.2024.1309509

**Published:** 2024-01-30

**Authors:** Di Wang, Xiaoshuang Wang, Yingyu Zhang, Le Yu, Jing An, Xiaodong Wang, Yue Huang, Xuemei Han

**Affiliations:** ^1^ Department of Neurology, China-Japan Union Hospital of Jilin University, Changchun, China; ^2^ Gastroenteric Medicine and Digestive Endoscopy Center, The Second Hospital of Jilin University, Changchun, Jilin, China; ^3^ Key Laboratory of Polymer Ecomaterials, Changchun Institute of Applied Chemistry, Chinese Academy of Sciences, Changchun, China

**Keywords:** interleukin 2, nanoparticles, CDK4/6 inhibitors, tumor immunotherapy, colon cancer

## Abstract

Immunotherapy of tumors plays a pivotal role in the current treatment of cancer. While interleukin 2 (IL-2) demonstrated its efficacy as an immunotherapeutic drug in the early days, its short blood circulation time poses challenges in maintaining effective therapeutic concentrations. Additionally, IL-2’s activation of regulatory T cells can counteract its anti-cancer effects. Therefore, the primary goal of this study was to formulate IL-2-carrying nanoparticles via boron-nitrogen coordination between methoxy poly (ethylene glycol) block poly-[(N-2-hydroxyethyl)-aspartamide]phenylboronic acid (mPEG-b-PHEA-PBA, P-PBA) and poly (L-lysine) (PLL). These nanoparticles are intended to be used in combination with CDK4/6 inhibitors to address the short blood circulation time of IL-2, reduce its immunosuppressive effects, and enhance the overall immune response. The envisaged outcome is a sustained and potent therapeutic effect, offering a novel and promising combination therapy strategy for tumor immunotherapy.

## Introduction

Interleukin 2 (IL-2), initially discovered over 40 years ago, was approved by the FDA in 1992 for the treatment of renal cell carcinoma as an anti-cancer immunotherapy (1). However, IL-2 requires frequent injections due to its short half-life, leading to various side effects (2). IL-2 is a multifunctional cytokine that regulates the immune response by binding to receptors with varying affinities (1, 3, 4). High-affinity receptors are predominantly expressed in Treg cells (5), while medium-affinity receptors are mainly expressed in CD8^+^ T cells (6). This necessitates high-dose IL-2 injections in anti-cancer treatments, but the activation of effector T cells coincides with the activation of immunosuppressive Tregs. In recent years, a variety of improvement schemes for IL-2 have swept through the market, such as IL-2 mutant (7), PEG-modified IL-2 (8), IL-2 immune complex (9), and IL-2-CD25 fusion protein (10). However, each approach has its limitations (11).

In this study, we employed a combination therapy to mitigate the immunosuppressive effects and induce a robust immune response. Specifically, we first utilized methoxy poly (ethylene glycol) block poly-[(N-2-hydroxyethyl)-aspartamide]phenylboronic acid (mPEG-*b*-PHEA-PBA, P-PBA) and poly (L-lysine) (PLL) to encapsulate IL-2 through boron-nitrogen coordination, forming IL-2-loaded polypeptide nanoparticles (P-IL-2), as previously reported (12). This formulation effectively prolonged the half-life of IL-2, and its ROS responsiveness facilitated drug release at the tumor site. Then, Palbociclib, a commonly used CDK4/6 inhibitor, was used for combination therapy with P-IL-2 (13, 14). CDK4/6 inhibitors, approved by the FDA in 2015 for the first-line treatment of HR(+)/HER2 (–) advanced breast cancer (15), primarily inhibit the phosphorylation of the retinoblastoma gene (Rb), blocking the G1 phase of the cell cycle in tumor cells (16). Later findings revealed additional mechanisms, including promoting tumor immunity, enhancing tumor antigen presentation, and inhibiting the proliferation of regulatory T cells (17–20). The results of our combination therapy demonstrated a significant reduction in Treg cells and a significant increase in CD8+ T cells in mice treated with P-IL-2 in combination with Palbociclib.

## Experimental

### Materials

Palbociclib was purchased from Shanghai Dibai Biological Technology CO. Ltd. Anhydrous *N*, *N*-dimethylformamide (DMF) and anhydrous dichloromethane were purchased from Energy Chemical (Shanghai, China). 4-dimethylaminopyridine (DMAP), Carbonyldiimidazole (CDI) and 4-(hydroxymethyl) phenylboronic acid pinacol ester were purchased from Energy Chemical (Shanghai, China). *N* ^ϵ^-Benzyloxycarbonyl-_L_-lysine-*N*-carboxyanhydride (Lys-NCA) and γ-Benzyl-_L_-aspartate-*N*-carboxyanhydride (BLA-NCA) were purchased from Chengdu Enlai Biological Technology CO. Ltd. (Chengdu, China). Other chemical reagents were purchased from Xilong Science Co., Ltd. (Shantou, China). Recombinant human IL-2 (IL-2) was provided by the Changchun Institute of Biological Products Co., Ltd. (Changchun, China). ELISA kits were purchased from Thermo Fisher Scientific (USA). The antibodies used for flow cytometry are listed in **Supplementary Table 1**.

### Characterization

^1^H nuclear magnetic resonance (^1^H NMR) spectra were recorded on a Bruker AV-300 NMR spectrometer in CDCl_3_, TFA-d, D_2_O, or DMSO-d_6_. The molecular weight was determined by gel permeation chromatography (GPC, SA). The size distribution of nanoparticles was determined by dynamic laser scattering (DLS) while their zeta potential was measured by a Zeta Potential/BI-90Plus particle size analyzer (Brookhaven Instruments Corporation, USA). The concentration of unloaded IL-2 was quantified by an IL-2 ELISA kit (Thermo Fisher, USA). Transmission electron microscope (TEM) images were obtained from the JEOL JEM-1011 (Tokyo, Japan). Fluorescence intensity was measured using a microplate reader (Bio-Rad, Hercules, CA, USA). Histological changes were observed with an optical microscope (Nikon Eclipse Ti, Optical Instruments Corporation, Ardmore, PA, USA). Lymphocytes were measured by using flow cytometry (BD FACS Celesta, USA). Using FlowJo software (Becton Dickinson and Company, USA), cell populations were identified and quantified.

### Cell lines and animals

The murine colorectal cancer cell lines CT26 were purchased from the BeNa Culture Collection (Beijing, China). Cells were cultured in RPMI 1640 medium supplemented with 10% FBS, 1% penicillin, and 1% streptomycin, and maintained at 37°C in a 5% CO_2_ atmosphere. BALB/c mice aged 6 to 8 weeks (female, average body weight: 16 to 18 g) were obtained from Beijing Vital River Laboratory Animal Technology Co., Ltd (Beijing, China). The Balb/c mice murine colorectal tumor model was established by injecting CT26 cells (1.0 × 10^6^ per mouse) subcutaneously into the right lower abdomen. All animal experimental procedures were conducted in accordance with the guidelines of the Jilin University Animal Care Facility and were approved by the Animal Ethics Committee of Jilin University.

### Synthesis of poly(L-lysine)

PLL was synthesized following a procedure described in our previous work (12, 21). Briefly, Lys-NCA (4.5 g) was dissolved in anhydrous DMF with n-hexamine (59.4 mg) and stirred at 35°C for 48 h. Poly (benzyloxycarbonyl-L-lysine) (PBLL) was obtained by precipitation with cold ether. Next, PBLL (2.25 g) was dissolved using 22.5 mL of trifluoroacetic acid, followed by a slow addition of 6.75 mL of HBr/acetic acid at 0°C and stirred for 1 h at 25°C. The product of cold ether precipitation was redissolved in DMF and dialyzed (MWCO=3500 Da) using water for 2 days. Finally, the white solid PLL was obtained by freeze-drying. The chemical structure of PLL was characterized using D_2_O as the solvent.

### Synthesis of methoxy poly(ethylene glycol) block poly-[(N-2-hydroxyethyl)-aspartamide] phenylboronic acid

The synthesis of P-PBA followed our established protocol (12, 21). Briefly, 4-(hydroxymethyl) phenylboronic acid pinacol ester (6.0 g, 1.0 equiv.) and carbonyldiimidazole (5.0 g, 1.2 equiv.) were dissolved in 60 ml of anhydrous dichloromethane and stirred for 3 h at 25°C. The reaction solution was then diluted with 150 mL of ethyl acetate, washed three times with saturated saline, three times with deionized water, and dried overnight with Na_2_SO_4_. The resulting white solid CDI-PBA was obtained by vacuum drying and its chemical structure was determined by ^1^H-NMR using CDCl_3_ as the solvent.

Subsequently, methoxy poly(ethylene glycol) block poly(γ-benzyl-L-aspartic acid) (mPEG-*b*-PBLA) was synthesized by the ring-opening polymerization reaction of BLA-NCA using mPEG-NH_2_ as an initiator. The mPEG-NH_2_ (Mn=5000 Da, 6.0 g) was dissolved in 6.0 mL of anhydrous DMF, and then BLA-NCA (9.6 g) and 120.0 mL of anhydrous dichloromethane were added to the above solution and stirred at 35°C for 3 days. Then acetic anhydride (1.2 g) was added to the above reaction solution and the reaction was continued for 12 h. White solid mPEG-*b*-PBLA was obtained by precipitation using cold ether. The structure of mPEG-*b*-PBLA was determined by ^1^H-NMR using TFA-d as the solvent.

Following this, mPEG-*b*-PBLA (11 g) and ethanolamine (6.0 g) were dissolved in 50.0 mL of anhydrous DMF and stirred at 35°C overnight. The product of cold ether precipitation was redissolved in DMF and dialyzed (MWCO=3500 Da) using water for 3 days. The white solid mPEG-*b*-PHEA was obtained by freeze-drying, and its structure was determined by ^1^H-NMR using TFA-d/D_2_O (1:9, *V:V*) as the solvent.

Finally, mPEG-*b*-PHEA (3.0 g), CDI-PBA (2.4 g), and DMAP (887 mg) were dissolved in 30.0 mL of anhydrous DMF and stirred at 50°C for 24 h. The product of cold ether precipitation was redissolved in DMF and dialyzed (MWCO=3500 Da) using water for 3 days. The P-PBA was obtained by freeze-drying and the structure was determined by ^1^H-NMR using DMSO-d_6_ as the solvent. GPC was also measured and the conditions were consistent with our previous work (12).

### Synthesis of P-IL-2

P-IL-2 was synthesized as per the procedures in a previous report (12), where the optimal ratio of P-PBA to IL-2 had been previously determined. Briefly, P-PBA (16.0 mg/mL) and IL-2 (1.0 mg/mL) were mixed and stirred to obtain P-PBA/IL-2, to which varying amounts of PLL were subsequently added and stirred on ice. The solution was then purified, washed, and the unloaded IL-2 was quantified. P-IL-2 was directly subjected to particle size and zeta potential measurements, and the image of P-IL-2 was obtained by TEM. The drug loading content (DLC) and drug loading efficiency (DLE) were calculated according to the following equations:


DLC=(weight of IL−2 loaded in nanoparticles)/(weight of IL−2−loaded nanoparticles)×100%

DLE=(weight of IL−2 loaded in nanoparticles)/(weight of feeding IL−2)×100%

### Stability of P-IL-2

P-IL-2 was dissolved in PBS buffer (pH 7.4). At predetermined time intervals (0.5, 1, 3, 6, and 24 h), 1.0 mL of the P-IL-2 solution was withdrawn, and the diameter was measured by dynamic laser scattering (DLS) to assess stability and dispersibility.

### ROS responsiveness of P-IL-2

P-IL-2 was dissolved in PBS buffer (pH 7.4) with or without 100.0 μM H_2_O_2_, and in PBS buffer (pH 7.4) with 10% FBS. The suspension was shaken for 8 h in a thermostatic incubator (37°C, 100 rpm). Subsequently, the diameter was then measured with DLS.

### Pharmacokinetics

The pharmacokinetics was characterized by measuring IL-2 levels through collecting the eye arterial blood of SD rats. Initially, six rats were randomly divided into two groups (n = 3) and treated with an intravenous injection of Cy5-labeled IL-2 or Cy5-labeled P-IL-2 (1.5 mg/kg based on IL-2). Prior to the pharmacokinetic experiment, both Cy5-labeled IL-2 and Cy5-labeled P-IL-2 concentrations were standardized to the same concentration (0.3 mg/mL) by UV-vis. Eye arterial blood was collected at specified times (0.25, 0.5, 1, 2, 4, 8, and 12 h) post-injection, heparinized, and then centrifuged at 2000g for 5 min to separate the serum. Subsequently, 100.0 μL of serum was added to each well, and the fluorescence intensity of each well was assessed using a microplate reader at the appropriate wavelength (λ_ex_ = 605 nm, λ_em_ = 665 nm). The half-life (t_1/2_) of the drug was calculated by non-compartmental analysis using PK solver.

### Anticancer efficiency *in vivo*

On Day -7, 1.0 × 10^6^ CT26 tumor cells were injected subcutaneously into the right lower abdomen of Balb/c mice to establish the CT26 tumor model. Once the tumor volume reached about 90 mm^3^, the mice were randomly divided into 6 groups (n = 5). Then they were treated with PBS, Palbociclib, IL-2, P-IL-2, IL-2+Palbociclib, and P-IL-2+Palbociclib. The initiation of treatment on Day 0 marked the beginning of the study. IL-2 (1.0 mg per kg) and P-IL-2 (1.0 mg per kg based on IL-2 concentration) were intravenously injected on Days 0, 3, 6, 9, and 12. Palbociclib (50 mg per kg, diluted in sterile water) was administered daily by oral gavage. Tumor size and body weight were recorded every other day. The tumor volume (*V*; mm^3^) was calculated as:


$${V\text{(mm}}^{2}{)=\text{the longest axis of tumors×}(\text{the shortest axis of tumors})}^{2}/2$$

The tumor suppression rate (TSR) was calculated as:


$$TSR\%=[(V_{C}-V_{X})/V_{C}]\times 100\%$$

Where *Vc* is the mean tumor volume in the PBS group and *Vx* represents the mean tumor volume in the other treatment groups.

### Immune analysis

Flow cytometry was conducted to analyze the levels of immune cells in tumors and spleens of mice subjected to different conditions. Flow cytometry analysis was carried out on Day 14 of the tumor inhibition experiment. After euthanizing the mice, spleens and tumors were harvested. Spleens were gently ground and filtered, with erythrocytes lysed using ACK lysis buffer. The tumor dissociation buffer was used to digest the tumor to obtain cell suspension, which was then filtered through 250 mesh nylon strainers. The resulting single-cell suspension was washed with PBS (0.01M, pH 7.4) containing 2% FBS, and stained for cell surface or nuclei using various fluorescence-conjugated antibodies targeting CD3, CD4, CD8, and FOXP3. The cells were fixed in 4% paraformaldehyde and flow cytometry was employed to detect the levels of CD8^+^ T cells, and FOXP3^+^ T cells in tumors and spleens. The collected data were analyzed using FlowJo software.

The assessment of cytokines within both tumor tissues and serum were meticulously conducted using enzyme-linked immunosorbent assay (ELISA) kits. In a concise, the harvested tumors underwent thorough washing with PBS (10 mM, pH 7.4) before being delicately sectioned into small, precisely measured pieces (20mg). The tumor tissue to make tissue homogenate. The resulting tumor homogenate was subjected to centrifugation at 3000 rpm for 10 minutes. The supernatant, carefully obtained post-centrifugation, underwent meticulous measurement utilizing ELISA kits, thereby revealing the precise content of TNF-α and IFN-γ. Simultaneously, the quantification of cytokines within the serum were performed directly using ELISA kits.

### H&E and TUNEL staining

On Day 14, mice were euthanized to assess histological changes in tumors and major organs. The heart, liver, spleen, lung, kidney, and tumors were collected, fixed in 4% buffered paraformaldehyde for 48 hours, and then processed into 5-μm-thick sections following standard protocols. These sections were stained with hematoxylin and eosin (H&E) or subjected to TUNEL immunofluorescence assays. Images capturing the histological alterations in the sections were obtained using a camera attached to an optical microscope.

### Systemic toxicity evaluation *in vivo*

Blood was collected from mice (n=5) after various treatments for the analysis of serum biochemical parameters. Liver function parameters (alanine aminotransferase -ALT-, aspartate aminotransferase -AST-, and alkaline phosphatase -AKP-) and kidney function parameters (creatinine -CRE-, uric acid -UA- and blood urea nitrogen -BUN-) were measured following the manufacturer’s instructions.

### Statistical analysis

Statistical analysis was carried out using GraphPad Prism8 for Windows (GraphPad Software, CA, USA). The significant difference between different groups was analyzed using an unpaired t-test. One-way ANOVA was used for multiple groups comparison followed by Turkey’s test. The data was presented as mean ± SD. *p*< 0.05 indicated a significant difference between groups.

## Results

### Characterization of PLL

PLL was synthesized according to the previously reported method (12, 21), and the synthesis route is shown in **Supplementary Figure 1A**. The ^1^H-NMR spectrum of PLL is shown in **Supplementary Figure 1B**. The characteristic peak of -CH- in the PLL main chain is represented by peak c at δ = 4.21 ppm, while peak a at δ = 0.75 ppm signifies the characteristic peak of -CH2- in the initiator. The area ratio of peak c to peak a was determined to be 25.39/3.00, indicating a degree of polymerization of 25 for PLL.

### Characterization of P-PBA

The ^1^H-NMR spectrum of CDI-PBA is presented in **Supplementary Figure 2**. As shown in **Supplementary Figure 3**, the area ratio of peak f at δ = 7.13 ppm to peak b at δ = 3.81 ppm in the ^1^H-NMR spectrum of mPEG-*b*-PBLA was about 95.5:452.0, indicating a polymerization degree of 19 for mPEG-*b*-PBLA. Subsequently, mPEG-*b*-PBLA was ammoniated with ethanolamine to obtain mPEG-*b*-PHEA. The ^1^H-NMR spectrum of mPEG-*b*-PHEA revealed the removal of the benzene ring of the side chain of mPEG-*b*-PBLA (**Supplementary Figure 4**). Finally, P-PBA was obtained through the reaction of CDI-PBA and mPEG-b-PHEA. In the ^1^H-NMR spectrum of P-PBA (**Supplementary Figure 5B**), the area ratio of peak h at δ = 5.13 ppm to peak c at δ = 4.62 ppm was 28.0:57.6, indicating a successful synthesis of P-PBA, with a grafting rate of phenylboronic at 24.3%. The *M*n of P-PBA, measured by GPC, was approximately 5.1 kDa (PDI = 1.19).

### Preparation and characterization of P-IL-2

P-PBA and PLL were successfully synthesized following previously reported procedures (12, 21). The optimal ratio of P-PBA to IL-2 was determined as described in previous reports (12). It has been established that boron-nitrogen coordination between the phenylboronic acid of P-PBA and the amino group in IL-2 can be successful and the optimal loading ratio has been determined. However, for the stability of the nanoparticles, it is necessary to continue incorporating PLL to obtain more compact IL-2 nanoparticles.

**Figure 1:**
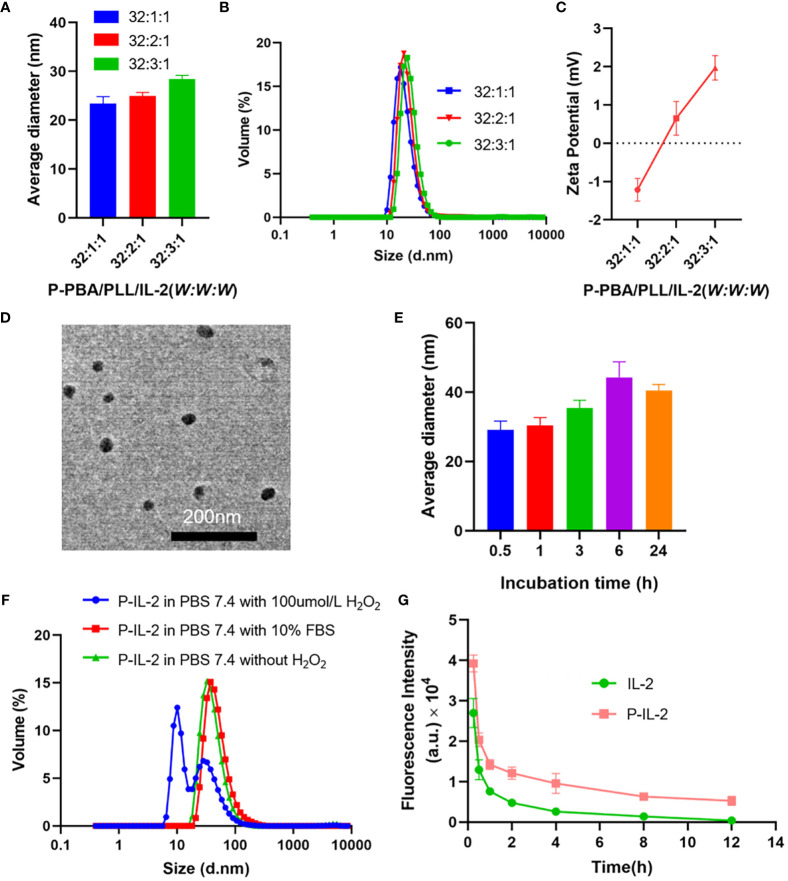
**(A, B)** Particle size of P-PBA/PLL/IL-2 with varying weight ratios, as determined by DLS. **(C)** Zeta potential of P-PBA/PLL/IL-2 with varying weight ratios. **(D)** TEM images for P-IL-2. Scale bar: 200 nm. **(E)** The particle size of P-IL-2 in PBS (0.01M, pH 7.4), measured by DLS at incubation of P-IL-2 (0.5h, 1h, 3h, 6h, 24h). **(F)** Particle size of P-IL-2 after 8h incubation in PBS (0.01M, pH 7.4) with or without H_2_O_2_, and PBS (0.01M, pH 7.4) with 10% FBS. **(G)** Pharmacokinetic results of IL-2 and P-IL-2.

To obtain the optimal particle size and zeta potential, different weight ratios of P-PBA/PLL/IL-2 (32:1:1, 32:2:1, 32:3:1) were selected for loading nanoparticles and characterized. DLE and DLC were calculated for each group and were found to be effectively loaded (**Supplementary Table 1**). Next, the characterization of particle size (**Figures 1A, B**) and zeta potential (**Figure 1C**) for each group was performed. The lowest potential was found at a weight ratio of 32:1:1 for P-PBA/PLL-IL-2 and the highest at a weight ratio of 32:3:1 (-1.2 ± 0.3 for 32:1:1; 0.7 ± 0.4 for 32:2:1; 2.0 ± 0.3 for 32:3:1). To obtain optimal results with a minimal synthesis ratio, the weight ratio of P-PBA/PLL/IL-2 was chosen to be 32:1:1. To visualize the particle size of P-IL-2 more closely, TEM was carried out on nanoparticles with a particle size of about 23.0 nm (**Figure 1D**).

### Stability of P-IL-2

As shown in **Figure 1E**, the particle size of P-IL-2 was measured at different time points (0.5, 1, 3, 6, and 24 h) after incubation in PBS (0.01 M, pH 7.4). There was no significant change in particle size up to 24 h, indicating good stability of P-IL-2.

### ROS responsiveness of P-IL-2

P-IL-2 was incubated in different solutions, including PBS buffer (pH 7.4) with or without 100 μmol/L H_2_O_2_, and PBS buffer (pH 7.4) with 10%FBS. The particle size of nanoparticles was measured 8h after incubation. As shown in **Figure 1F**, the particle size of P-IL-2 did not change significantly in PBS buffer (pH 7.4) without 100 μmol/L H_2_O_2_ or PBS buffer (pH 7.4) with 10% FBS. However, there was a significant change in the presence of H_2_O_2_, indicating that P-IL-2 is ROS-responsive.

### Pharmacokinetics

Cy5-labeled IL-2 and cy5-labeled P-IL-2 were intravenously injected into each group of SD rats, and fluorescence intensity was measured by collecting eye arterial blood at different time points after injection to calculate the drug half-life. As shown in **Figure 1G**, the half-life of P-IL-2 (*t*_1/2_ = 8.8 ± 1.4 h) was 3.14-fold prolonged compared to the half-life of IL-2 (*t*_1/2_ = 2.8 ± 0.2 h).

### Anticancer efficiency *in vivo*

**Figure 2:**
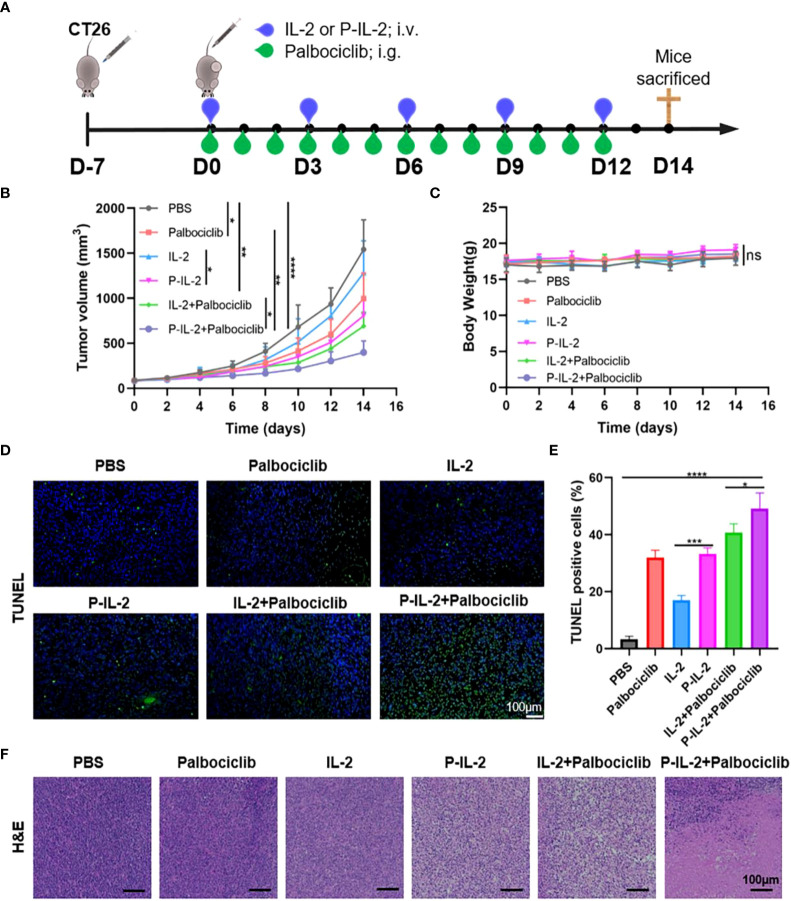
Anti-cancer effect *in vivo* **(A)** The schematic of CT26 tumor modeling and treatment protocols. **(B)** Tumor growth curves for all groups treated by various therapy schedules. **(C)** Body weight changes for all groups treated by various therapy schedules. **(D)** TUNEL immunofluorescence detection of tumors after varying treatments. **(E)** Proportion of TUNEL positive cells. **(F)** H&E staining of tumors after various treatments. Scale bar: 100 µm. ns: no significance, *: p< 0.05; **: p<0.01, ***: p<0.001, ****: p<0.0001.

As previously described, P-IL-2 with an extended half-life was successfully synthesized, and we further compared the antitumor efficiency with the combination of Palbociclib and P-IL-2 *in vivo* (**Figure 2A**). As shown in **Figure 2B**, compared with the PBS-treated group, the Palbociclib-treated group had a TSR of 35.29%, whereas the IL-2 group did not exhibit a significant anti-tumor effect. However, when IL-2 was combined with Palbociclib, the TSR increased to 55.25%. The increase in TSR to 47.67% in the P-IL-2-treated group compared to the IL-2-treated group may be attributed to the longer half-life of P-IL-2, allowing its continuous efficacy in the body. The TSR in the P-IL-2 and Palbociclib combination group was 74.08%, with a 1.34-fold increase in tumor suppression and a 1.73-fold reduction in tumor volume compared to the IL-2 and Palbociclib combination group. Importantly, no significant weight loss was observed in mice on any of the treatment regimens (**Figure 2C**).

To further validate the tumor suppressive effect of the drug, tumor tissue was also obtained at the end of treatment for H&E and TUNEL staining (**Figures 2D–F**). In the PBS-treated group, the tumor had a large nucleus and abundant cytoplasm in H&E staining, and there was essentially no apoptosis in TUNEL staining, suggesting that the tumor had a strong proliferative capacity. In contrast, large swathes of cell necrosis and apoptosis were observed in the P-IL-2 and Palbociclib combination treatment group. This observation underscores the superior therapeutic effect of the combination of P-IL-2 and Palbociclib.

### Immune analysis

To further elucidate the anti-tumor effect of the combination treatment of P-IL-2 and Palbociclib, spleens and tumor tissues were subjected to flow cytometry measurements to observe the changes in the tumor immune microenvironment.

**Figure 3:**
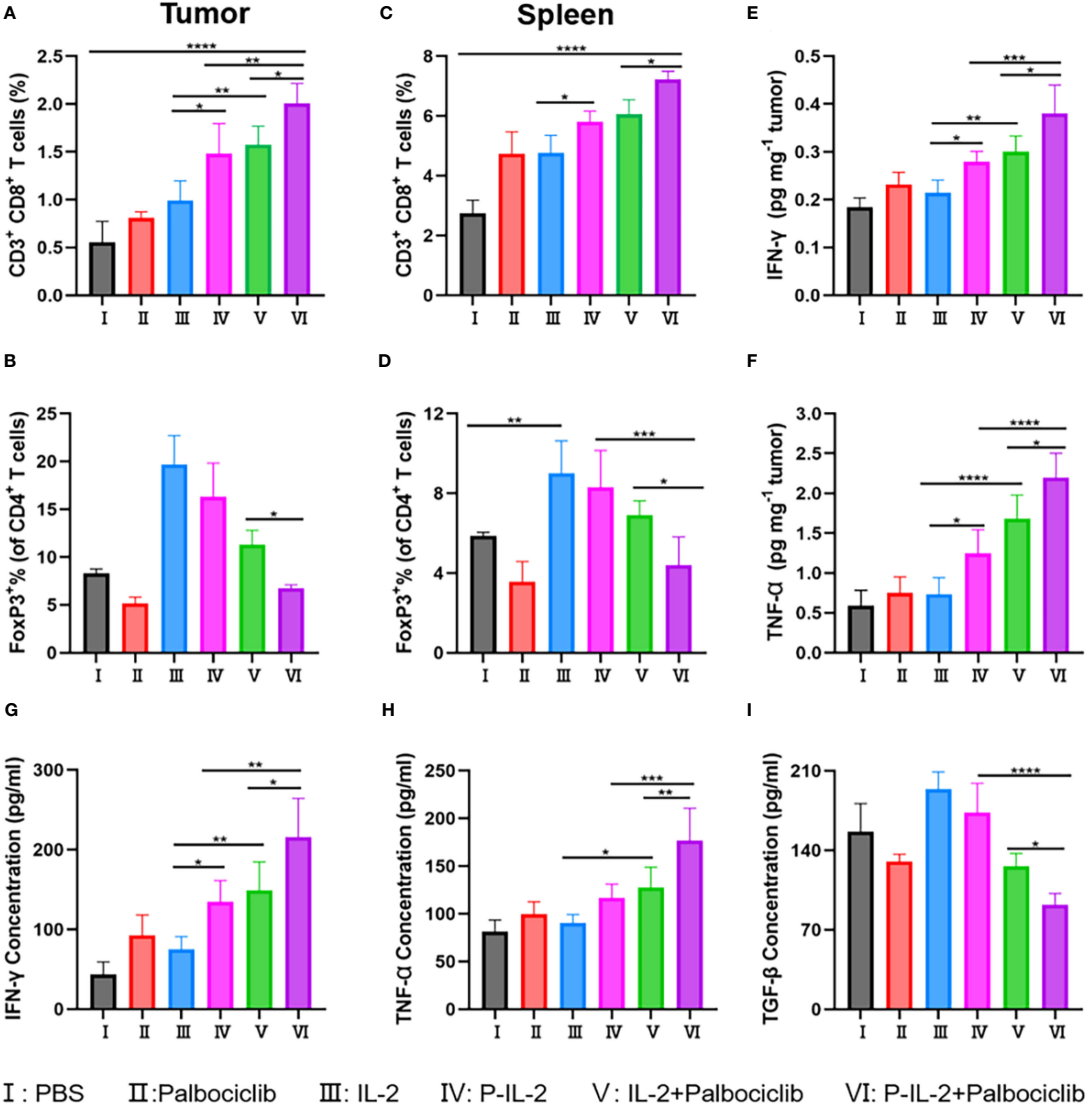
Immune analysis results after different treatments. **(A, B)** The relative quantification of CD8^+^ T cells and Treg cells in tumors. **(C, D)** The relative quantification of CD8^+^ T cells and Treg cells in spleens. **(E)** IFN-γ and **(F)** TNF-α in the tumors after various treatments. Concentration of **(G)** IFN-γ, **(H)** TNF-α and **(I)** TGF-β in the serum after various treatments. *: p<0.05, **: p<0.01, ***: p<0.001, ****: p<0.0001.

Specifically, CD8^+^ T cells were significantly higher in the P-IL-2-treated group compared to the PBS-treated or IL-2-treated groups. This suggests a prolonged blood circulation time for P-IL-2, allowing the drug to continue working and stimulating the activation of effector T cells, thereby enhancing the anti-tumor effect. When compared with the P-IL-2-treated group, CD8^+^ T cells were further elevated in the P-IL-2 and Palbociclib combination treatment group. This demonstrates that the combined treatment of Palbociclib and IL-2 works together to activate CD8^+^ T cells with a stronger immune response, suggesting that such a combination strategy can produce better immune responses and anti-tumor effects (**Figures 3A, C**). Treg cells were reduced in both spleen and tumor in the combination treatment group compared to the IL-2 treatment group or the P-IL-2 treatment group (**Figures 3B, D**). Such a reduction indicates that the inhibitory effect of Palbociclib on Treg cells antagonized the activating effect of IL-2 on Treg cells, thus further enhancing the tumor effect by improving the immune microenvironment.

In order to better verify the immune effect, cytokines were also detected. Activated CD8^+^ T cells secrete IFN-γ and TNF-α, initiating a cascade of inhibitory immune responses against the tumor. Conversely, TGF-β hinders pro-inflammatory cells and Th cells while promoting Treg cells. Illustrated in **Figures 3E–I**, is an escalation in IFN-γ and TNF-α secretion post-combination therapy, indicating a heightened immune response of CD8^+^ T cells. Additionally, the observed decline in TGF-β suggests an impact on the development or function of Treg cells. Integrating these findings with the flow cytometry analysis of CD8^+^ T cells and Treg cells within the tumor locale, it is deduced that the combination treatment group effectively reversed the tumor microenvironment compared to the IL-2 alone treatment group.

### H&E staining of main organs

**Figure 4:**
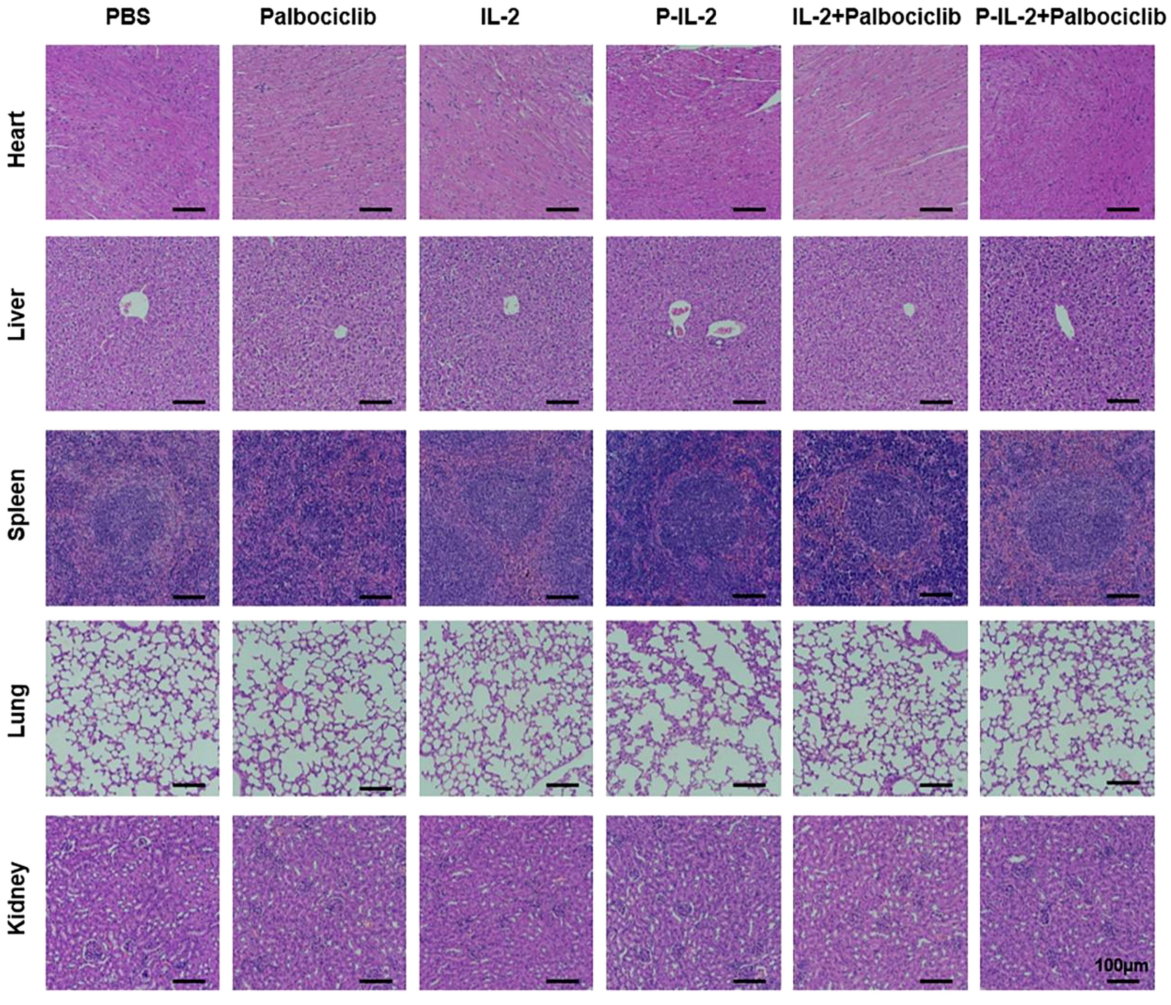
H&E staining of mice organs after varying treatments. Scale bar: 100μm.

Major organs were taken and analyzed for H&E staining to visualize the presence of pathological changes. As illustrated in **Figure 4**, no significant pathological changes were found in the vital organs of mice after various treatments.

### Systemic toxicity evaluation *in vivo*

**Figure 5:**
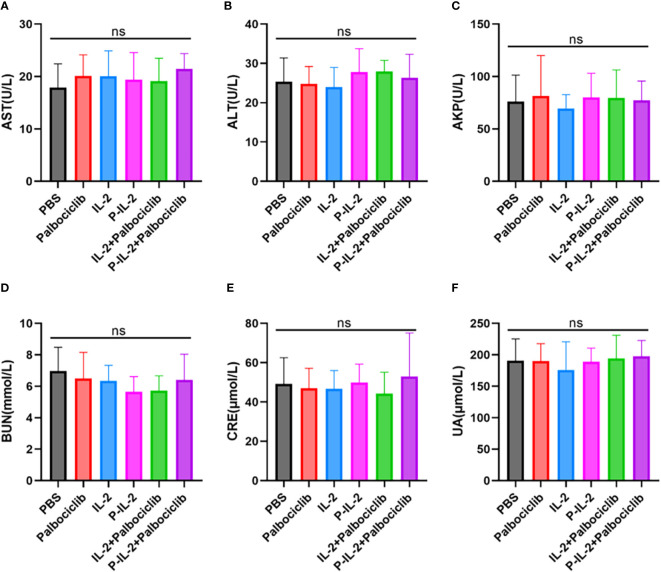
Indicators of liver and kidney function after treatment in all groups. **(A)** AST. **(B)** ALT. **(C)** AKP. **(D)** BUN. **(E)** CRE. **(F)** UA. ns, no significance.

Liver (AST: **Figure 5A**; ALT: **Figure 5B**; AKP: **Figure 5C**) and kidney (BUN: **Figure 5D**; CRE: **Figure 5E**: UA: **Figure 5F**) function indices of mice were examined, and there was no significant difference between the groups. Together with the H&E staining results, the drugs were shown to have no significant systemic toxicity.

## Discussion

IL-2 was identified as an early potent immunotherapeutic agent against cancer (22), demonstrating different affinities to various receptors (23). At low doses, it binds to high-affinity receptors, activating regulatory T cells and exerting immunosuppressive effects suitable for treating autoimmune diseases (24). At high doses, IL-2 binds to medium-affinity receptors, activating effector T cells and exerting immune-activating effects, which are employed in anti-tumor therapy (25). In anti-tumor therapy with high-dose IL-2, activation of effector T cells is accompanied by activation of regulatory T cells, leading to immune activation (26). However, its short half-life has limited its clinical application (27, 28). In recent years, IL-2 research has been reinvigorated in an attempt to have better therapeutic efficacy with fewer toxic side effects by improving IL-2 preparations (29–31).

In this study, IL-2 was loaded by boron and nitrogen coordination to form nanoparticles, and the optimal ratio of P-PBA/PLL/IL-2 was screened to satisfy both the particle size potential and minimal immunogenicity. Based on the characterization results, P-PBA/PLL/IL-2 (*W: W: W*) =32:1:1 was selected. This formulation demonstrated good stability and responsiveness, consistent with the results of previous studies (12). The pharmacokinetic experiments confirmed a 3.14-fold extension in the half-life of P-IL-2, indicating prolonged circulation in the bloodstream. Moreover, the tumor site exhibited a high level of reactive oxygen species, and P-IL-2’s responsiveness to ROS facilitated selective release at the tumor site, thereby enhancing the anti-tumor effect.

**Figure 6:**
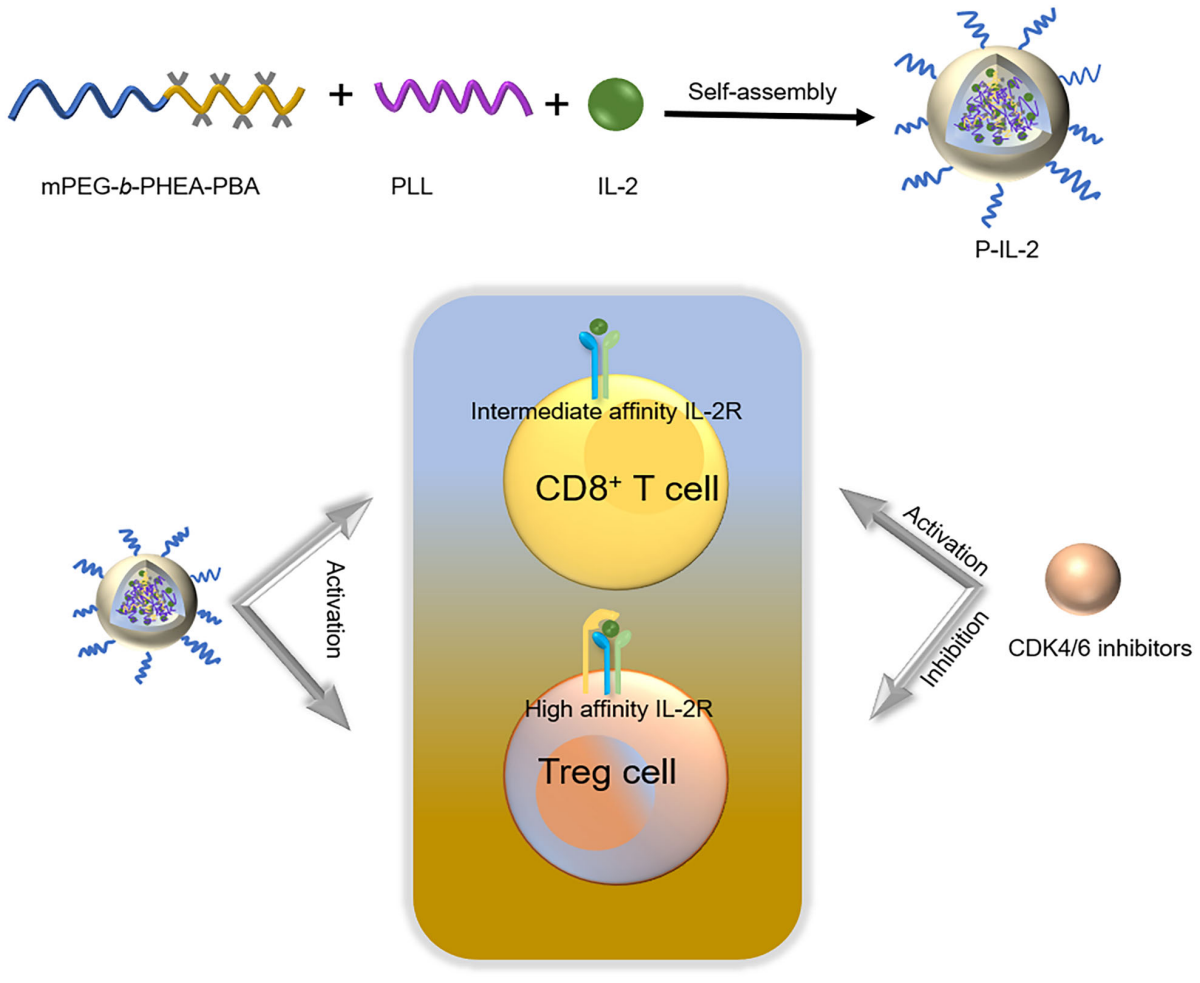
Scheme of P-IL-2 preparation and the combined mechanism of P-IL-2 and CDK4/6 inhibitors.

CDK4/6 inhibitors, like Palbociclib, exert their anti-tumor effects by maintaining the retinoblastoma gene (Rb) in a non-phosphorylated state, leading to cell cycle arrest, cell senescence, apoptosis, and enhanced immunogenicity (17, 32, 33). These inhibitors affect the tumor immune microenvironment by inhibiting the proliferation of regulatory T cells and enhancing the activation of effector T cells, in addition to their direct effects on tumor cells (34). Flow Cytometry in this work confirmed Palbociclib’s effect on the immune microenvironment, aligning with previous findings (17). We harnessed its impact on the tumor immune microenvironment and combined it with IL-2 to counteract IL-2’s immunosuppressive effect, aiming to enhance the immune response and improve the anti-tumor effect without increased toxicity. The combined mechanism of P-IL-2 and CDK4/6 inhibitors was shown in **Figure 6**.

The CT26 mouse model was selected to validate the effectiveness of CDK4/6 inhibitors and IL-2 treatment. Compared to IL-2-treated mice, P-IL-2-treated mice exhibited slowed tumor growth, as well as a relative decrease in regulatory T cells and a relative increase in CD8^+^ T cells in the immune microenvironment. This further demonstrates the superior immunotherapeutic effect of P-IL-2. Despite the activation of regulatory T cells, we combined it with CDK4/6 inhibitors (Palbociclib) to enhance the immune response while reversing its immunosuppressive effects, resulting in further slowed tumor growth. Pathologically, numerous necrotic and apoptotic cancer cells were observed. At the cytological level, the reduction of Treg cells and the increase in CD8+ T cells underscored the reversal of the immunosuppressive effect of the tumor microenvironment. Furthermore, activated CD8^+^ T cells secrete IFN-γ and TNF-α, initiating a cascade of inhibitory immune responses against the tumor. Conversely, TGF-β hinders pro-inflammatory cells and Th cells while promoting Treg cells (35, 36). Illustrated in **Figures 3E–I**, is an escalation in IFN-γ and TNF-α secretion post-combination therapy, indicating a heightened immune response of CD8^+^ T cells. Meanwhile, the observed decline in TGF-β suggests an impact on the development or function of Treg cells. The levels of proinflammatory cytokines IFN-γ and TNF-α, exhibited elevation, while the anti-inflammatory factor TGF-β demonstrated a modest decrease upon the scrutiny of cytokine levels in both the tumor and serum. Integrating these findings with the flow cytometry analysis of CD8^+^ T cells and Treg cells within the tumor locale, it is deduced that the combination treatment group effectively reversed the tumor microenvironment. This combined treatment strategy continued to promote the proliferation of CD8^+^ T cells in tumor areas, effectively inducing an immune response and obtaining a better suppression of the immune response. response, effectively inducing an immune response and achieving superior tumor suppression. This may be attributed to the dual action of CDK4/6 inhibitors inhibiting Treg cells and activating Teff cells (37–39). Ensuring both therapeutic efficacy and biosafety, this study observed the main organs at the pathological level, detecting no significant lesions or metastases. Serum analysis for liver and kidney function markers revealed no significant changes, collectively proving the biological safety of the combined treatment.

In conclusion, this study synthesized IL-2 nanoparticles and combined them with CDK4/6 inhibitors (Palbociclib), resulting in superior tumor suppression in mice without increased toxicity. These findings encourage further exploration of the clinical application of IL-2 and propose a new combination therapy paradigm that could reshape our approach to cancer immunotherapy.

## Data availability statement

The original contributions presented in the study are included in the article/**Supplementary Material**, further inquiries can be directed to the corresponding authors.

## Ethics statement

The animal study was approved by Jilin University Animal Care Facility. The study was conducted in accordance with the local legislation and institutional requirements.

## Author contributions

DW: Conceptualization, Writing – original draft, Investigation, Writing – review & editing. XSW: Writing – review & editing, Data curation, Software. YZ: Writing – review & editing, Supervision. LY: Supervision, Writing – review & editing, Formal analysis. JA: Writing – original draft. XDW: Writing – original draft, Supervision. YH: Writing – original draft, Conceptualization. XH: Writing – original draft, Writing – review & editing.

## References

[1] Raker VK Becker C Landfester K Steinbrink K. Targeted activation of T cells with IL-2-coupled nanoparticles. Cells (2020) 9(9):2063. doi: 10.3390/cells9092063 32917054 PMC7565705

[2] Liu F Ul Amin T Liang DP Park MS Alhamadsheh MM. Enhancing the pharmacokinetic profile of interleukin 2 through site-specific conjugation to a selective small-molecule transthyretin ligand. J Med Chem (2021) 64(19):14876–86. doi: 10.1021/acs.jmedchem.1c01426 PMC9625839 34542267

[3] Solomon I Amann M Goubier A Arce Vargas F Zervas D Qing C. CD25-T(reg)-depleting antibodies preserving IL-2 signaling on effector T cells enhance effector activation and antitumor immunity. Nat Cancer (2020) 1(12):1153–66. doi: 10.1038/s43018-020-00133-0 PMC7116816 33644766

[4] Su EW Moore CJ Suriano S Johnson CB Songalia N Patterson A. IL-2Rα mediates temporal regulation of IL-2 signaling and enhances immunotherapy. Sci Transl Med (2015) 7(311):311ra170. doi: 10.1126/scitranslmed.aac8155 PMC4805116 26511507

[5] Hashimoto M Araki K Cardenas MA Li P Jadhav RR Kissick HT. PD-1 combination therapy with IL-2 modifies CD8(+) T cell exhaustion program. Nature (2022) 610(7930):173–81. doi: 10.1038/s41586-022-05257-0 PMC9793890 36171288

[6] Orozco Valencia A Camargo Knirsch M Suavinho Ferro E Antonio Stephano M. Interleukin-2 as immunotherapeutic in the autoimmune diseases. Int Immunopharmacol (2020) 81:106296. doi: 10.1016/j.intimp.2020.106296 32058934

[7] Gaggero S Martinez-Fabregas J Cozzani A Fyfe PK Leprohon M Yang J. IL-2 is inactivated by the acidic pH environment of tumors enabling engineering of a pH-selective mutein. Sci Immunol (2022) 7(78):eade5686. doi: 10.1126/sciimmunol.ade5686 36459543

[8] Ptacin JL Caffaro CE Ma L San Jose Gall KM Aerni HR Acuff NV. An engineered IL-2 reprogrammed for anti-tumor therapy using a semi-synthetic organism. Nat Commun (2021) 12(1):4785. doi: 10.1038/s41467-021-24987-9 34373459 PMC8352909

[9] VanDyke D Iglesias M Tomala J Young A Smith J Perry JA. Engineered human cytokine/antibody fusion proteins expand regulatory T cells and confer autoimmune disease protection. Cell Rep (2022) 41(3):111478. doi: 10.1016/j.celrep.2022.111478 36261022 PMC9631798

[10] Hernandez R LaPorte KM Hsiung S Santos Savio A Malek TR. High-dose IL-2/CD25 fusion protein amplifies vaccine-induced CD4(+) and CD8(+) neoantigen-specific T cells to promote antitumor immunity. J Immunother Cancer (2021) 9(9):e002865. doi: 10.1136/jitc-2021-002865 34475132 PMC8413969

[11] Hernandez R Põder J LaPorte KM Malek TR. Engineering IL-2 for immunotherapy of autoimmunity and cancer. Nat Rev Immunol (2022) 22(10):614–28. doi: 10.1038/s41577-022-00680-w 35217787

[12] Wang XS Zheng ZS Zheng MF Wang D Zhang HL Zhang ZQ. IL-2-loaded polypeptide nanoparticles for enhanced anti-cancer immunotherapy. Chin J POLYM Sci (2023) 41(7):1059–68. doi: 10.1007/s10118-023-2898-2

[13] Liu ZL Yu HY Shen N Tang ZH Chen XS. A ROS-stimulus-responsive nanocarrier loading with guanidine-modified hydroxycamptothecin prodrug for enhanced anti-tumor efficacy. Ccs Chem (2020) 2(5):305–16. doi: 10.31635/ccschem.020.202000133

[14] Liu ZL Shen N Tang ZH Zhang DW Ma LL Yang CG. An eximious and affordable GSH stimulus-responsive poly(α-lipoic acid) nanocarrier bonding combretastatin A4 for tumor therapy. Biomater Sci (2019) 7(7):2803–11. doi: 10.1039/c9bm00002j 31062006

[15] Yuan K Wang X Dong H Min W Hao H Yang P. Selective inhibition of CDK4/6: A safe and effective strategy for developing anticancer drugs. Acta Pharm Sin B (2021) 11(1):30–54. doi: 10.1016/j.apsb.2020.05.001 33532179 PMC7838032

[16] Álvarez-Fernández M Malumbres M. Mechanisms of sensitivity and resistance to CDK4/6 inhibition. Cancer Cell (2020) 37(4):514–29. doi: 10.1016/j.ccell.2020.03.010 32289274

[17] Goel S DeCristo MJ Watt AC BrinJones H Sceneay J Li BB. CDK4/6 inhibition triggers anti-tumour immunity. Nature (2017) 548(7668):471–5. doi: 10.1038/nature23465 PMC5570667 28813415

[18] Lelliott EJ Kong IY Zethoven M Ramsbottom KM Martelotto LG Meyran D. CDK4/6 inhibition promotes antitumor immunity through the induction of T-cell memory. Cancer Discovery (2021) 11(10):2582–601. doi: 10.1158/2159-8290.CD-20-1554 33990344

[19] Deng J Wang ES Jenkins RW Li S Dries R Yates K. CDK4/6 inhibition augments antitumor immunity by enhancing T-cell activation. Cancer Discovery (2018) 8(2):216–33. doi: 10.1158/2159-8290.Cd-17-0915 PMC5809273 29101163

[20] Heckler M Ali LR Clancy-Thompson E Qiang L Ventre KS Lenehan P. Inhibition of CDK4/6 promotes CD8 T-cell memory formation. Cancer Discovery (2021) 11(10):2564–81. doi: 10.1158/2159-8290.Cd-20-1540 PMC8487897 33941591

[21] Lv S Tang Z Li M Lin J Song W Liu H. Co-delivery of doxorubicin and paclitaxel by PEG-polypeptide nanovehicle for the treatment of non-small cell lung cancer. Biomaterials (2014) 35(23):6118–29. doi: 10.1016/j.biomaterials.2014.04.034 24794923

[22] Overwijk WW Tagliaferri MA Zalevsky J. Engineering IL-2 to give new life to T cell immunotherapy. Annu Rev Med (2021) 72:281–311. doi: 10.1146/annurev-med-073118-011031 33158368

[23] Leonard WJ Lin JX. Strategies to therapeutically modulate cytokine action. Nat Rev Drug Discovery (2023) 22(10):827–54. doi: 10.1038/s41573-023-00746-x 37542128

[24] Lykhopiy V Malviya V Humblet-Baron S Schlenner SM. IL-2 immunotherapy for targeting regulatory T cells in autoimmunity. Genes Immun (2023) 24(5):248–62. doi: 10.1038/s41435-023-00221-y PMC10575774 37741949

[25] Lopes JE Fisher JL Flick HL Wang C Sun L Ernstoff MS. ALKS 4230: a novel engineered IL-2 fusion protein with an improved cellular selectivity profile for cancer immunotherapy. J Immunother Cancer (2020) 8(1):e000673. doi: 10.1136/jitc-2020-000673 32317293 PMC7204809

[26] Sun Z Ren Z Yang K Liu Z Cao S Deng S. A next-generation tumor-targeting IL-2 preferentially promotes tumor-infiltrating CD8(+) T-cell response and effective tumor control. Nat Commun (2019) 10(1):3874. doi: 10.1038/s41467-019-11782-w 31462678 PMC6713724

[27] Shi L Sheng J Chen G Zhu P Shi C Li B. Combining IL-2-based immunotherapy with commensal probiotics produces enhanced antitumor immune response and tumor clearance. J Immunother Cancer (2020) 8(2):e000973. doi: 10.1136/jitc-2020-000973 33028692 PMC7542661

[28] Sharma M Khong H Fa'ak F Bentebibel SE Janssen LME Chesson BC. Bempegaldesleukin selectively depletes intratumoral Tregs and potentiates T cell-mediated cancer therapy. Nat Commun (2020) 11(1):661. doi: 10.1038/s41467-020-14471-1 32005826 PMC6994577

[29] Bhuiyan AM Dougan M. Engineering T cell memory for antitumor immunity. Trends Pharmacol Sci (2022) 43(1):1–3. doi: 10.1016/j.tips.2021.11.003 34785086

[30] Mo F Yu Z Li P Oh J Spolski R Zhao L. An engineered IL-2 partial agonist promotes CD8(+) T cell stemness. Nature (2021) 597(7877):544–8. doi: 10.1038/s41586-021-03861-0 PMC9172917 34526724

[31] Codarri Deak L Nicolini V Hashimoto M Karagianni M Schwalie PC Lauener L. PD-1-cis IL-2R agonism yields better effectors from stem-like CD8(+) T cells. Nature (2022) 610(7930):161–72. doi: 10.1038/s41586-022-05192-0 PMC9534752 36171284

[32] Finn RS Dering J Conklin D Kalous O Cohen DJ Desai AJ. PD 0332991, a selective cyclin D kinase 4/6 inhibitor, preferentially inhibits proliferation of luminal estrogen receptor-positive human breast cancer cell lines in *vitro* . Breast Cancer Res (2009) 11(5):R77. doi: 10.1186/bcr2419 19874578 PMC2790859

[33] Vijayaraghavan S Karakas C Doostan I Chen X Bui T Yi M. CDK4/6 and autophagy inhibitors synergistically induce senescence in Rb positive cytoplasmic cyclin E negative cancers. Nat Commun (2017) 8:15916. doi: 10.1038/ncomms15916 28653662 PMC5490269

[34] Bai X Guo ZQ Zhang YP Fan ZZ Liu LJ Liu L. CDK4/6 inhibition triggers ICAM1-driven immune response and sensitizes LKB1 mutant lung cancer to immunotherapy. Nat Commun (2023) 14(1):1247. doi: 10.1038/s41467-023-36892-4 36871040 PMC9985635

[35] Wang M Hu DR Yang Y Shi K Li JA Liu QY. Enhanced chemo-immunotherapy strategy utilizing injectable thermosensitive hydrogel for the treatment of diffuse peritoneal metastasis in advanced colorectal cancer. Adv Sci (2023) 10(35):e2303819. doi: 10.1002/advs.202303819 PMC10724414 37875399

[36] Li X Zhou QH Japir AMM Dutta D Lu NN Ge ZS. Protein-delivering nanocomplexes with fenton reaction-triggered cargo release to boost cancer immunotherapy. ACS Nano (2022) 16(9):14982–99. doi: 10.1021/acsnano.2c06026 36017992

[37] Scirocchi F Scagnoli S Botticelli A Di Filippo A Napoletano C Zizzari IG. Immune effects of CDK4/6 inhibitors in patients with HR(+)/HER2(-) metastatic breast cancer: Relief from immunosuppression is associated with clinical response. EBioMedicine (2022) 79:104010. doi: 10.1016/j.ebiom.2022.104010 35477069 PMC9061627

[38] Nayyar N de Sauvage MA Chuprin J Sullivan EM Singh M Torrini C. CDK4/6 inhibition sensitizes intracranial tumors to PD-1 blockade in preclinical models of brain metastasis. Clin Cancer Res (2023) 30(2):420–35. doi: 10.1158/1078-0432.Ccr-23-0433 PMC10872577 37611074

[39] Hernandez R Toomer KH Põder J Santos Savio A Hsiung S Malek TR. Sustained IL-2R signaling of limited duration by high-dose mIL-2/mCD25 fusion protein amplifies tumor-reactive CD8(+) T cells to enhance antitumor immunity. Cancer Immunol Immunother (2021) 70(4):909–21. doi: 10.1007/s00262-020-02722-5 PMC7979461 33037893

