# Supplementary material for: The combination of IL-2 nanoparticles and Palbociclib enhances the anti-tumor immune response for colon cancer therapy
Source: Front Immunol. 2024 Jan 30;15:1309509. doi: 10.3389/fimmu.2024.1309509 (PMC10861758; doi:10.3389/fimmu.2024.1309509)
Supplement: Supplementary file 1 [file Presentation_1.pdf]

Supplementary Material

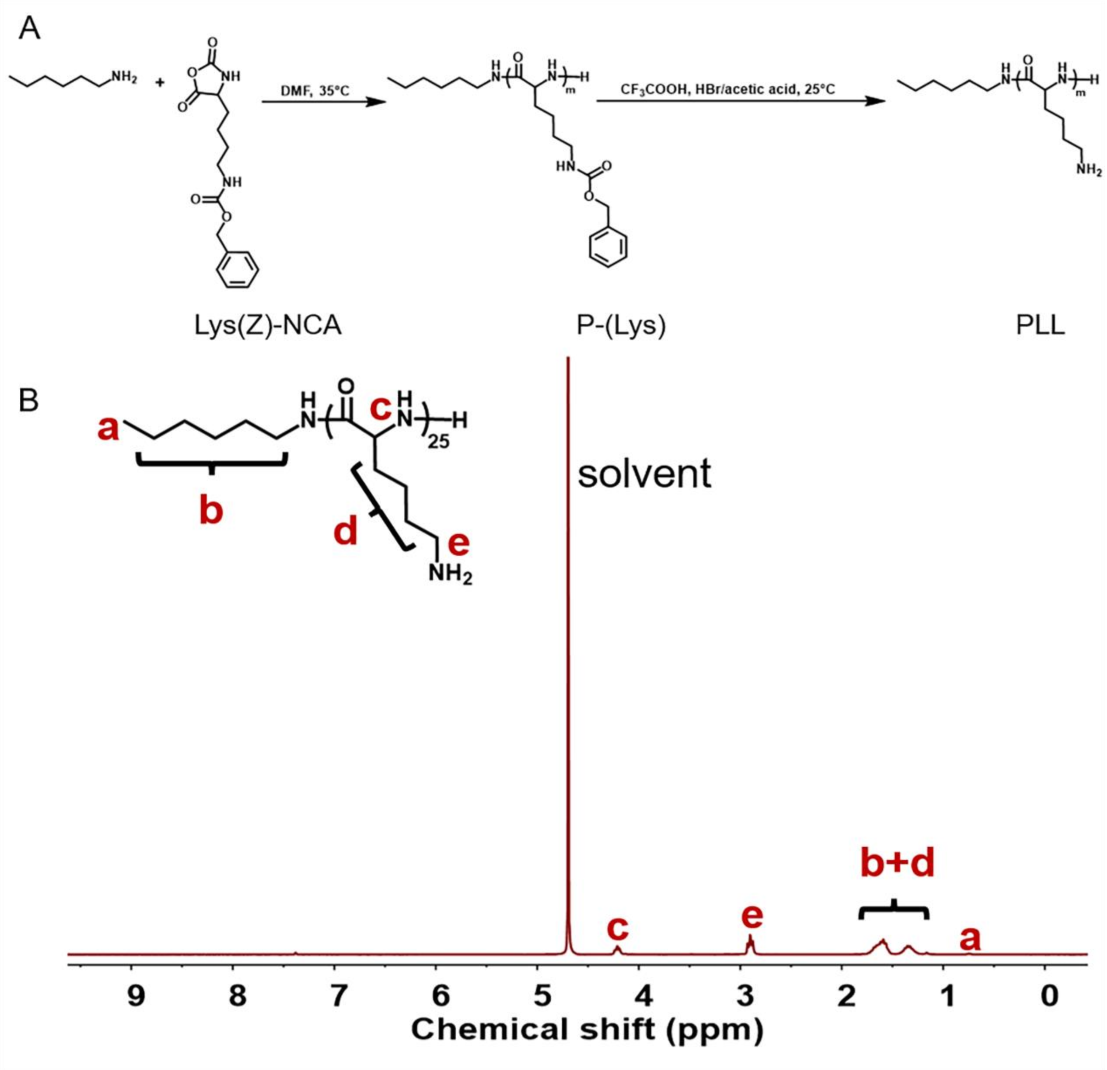

Figure S1 (A)Synthesized route of poly(L-lysine) (PLL). (B)^{1}H NMR spectrum of poly(L-lysine) (PLL).

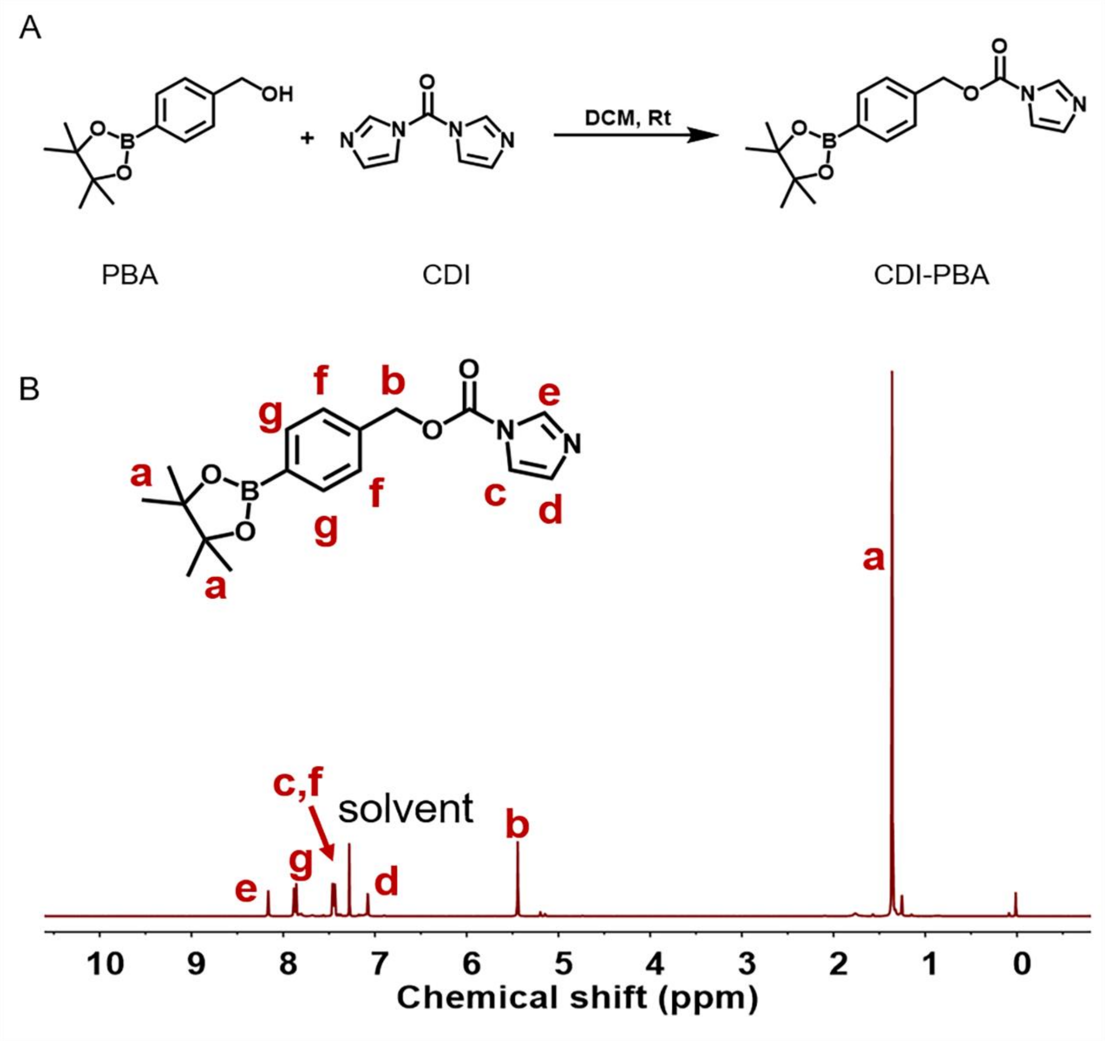

Figure S2 (A)Synthesized route of carbonyldiimidazole-phenylboronic acid (CDI-PBA). (B)^{1}H NMR spectrum of carbonyldiimidazole-phenylboronic acid (CDI-PBA).

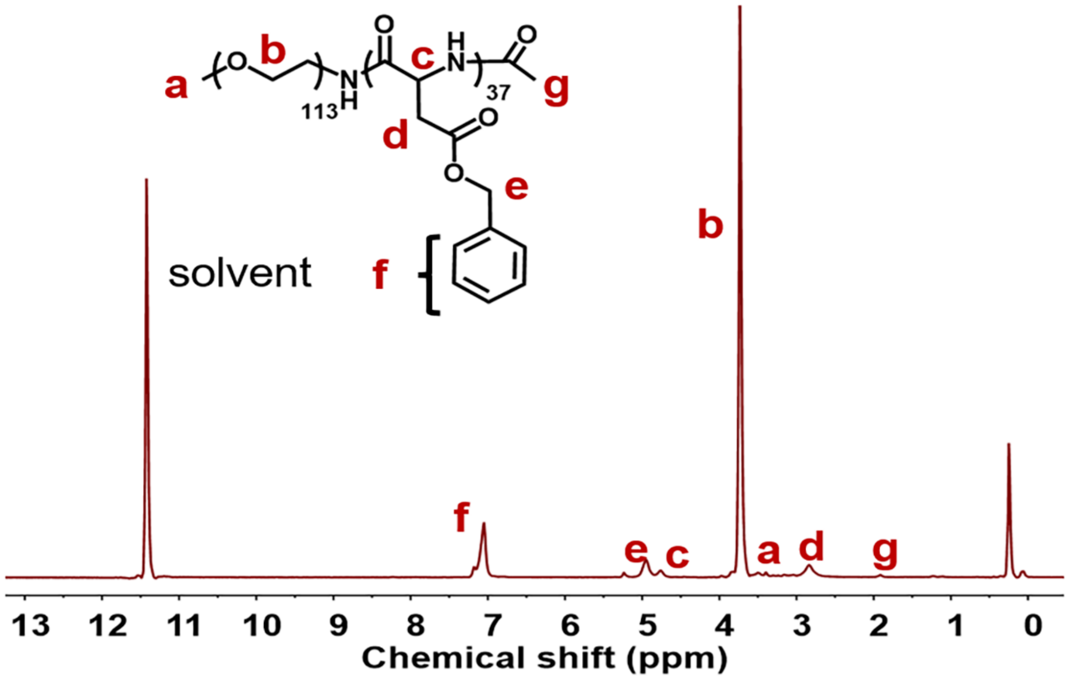

Figure S3 ^{1}H NMR spectrum of methoxy poly(ethylene glycol) block poly(γ-benzyl-L-aspartate) (mPEG-*b*-PBLA).

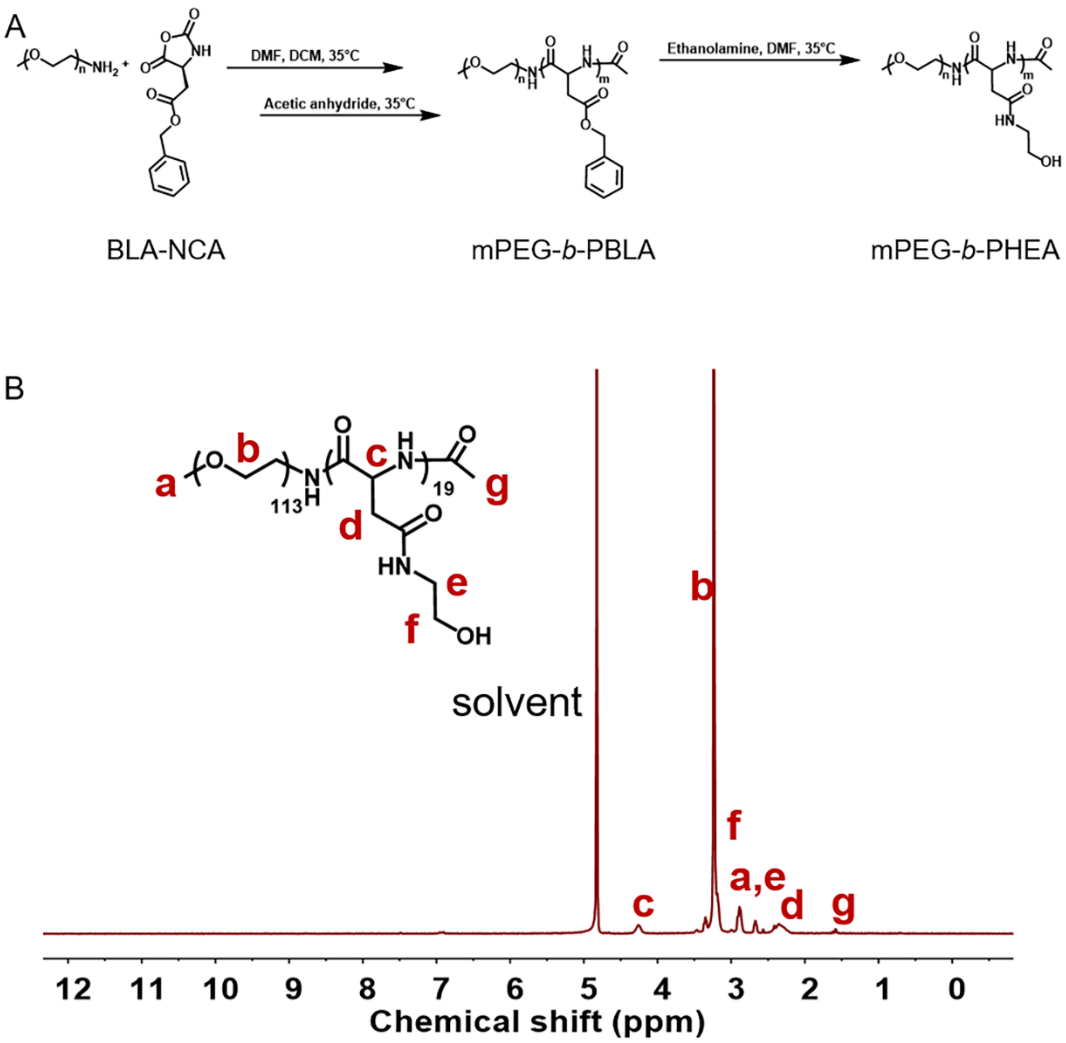

Figure S4 (A)Synthesized route of methoxy poly(ethylene glycol) block poly-[(N-2-hydroxyethyl)-aspartamide] (mPEG-*b*-PHEA). (B) ^{1}H NMR spectrum of methoxy poly(ethylene glycol) block poly-[(N-2-hydroxyethyl)-aspartamide] (mPEG-*b*-PHEA).

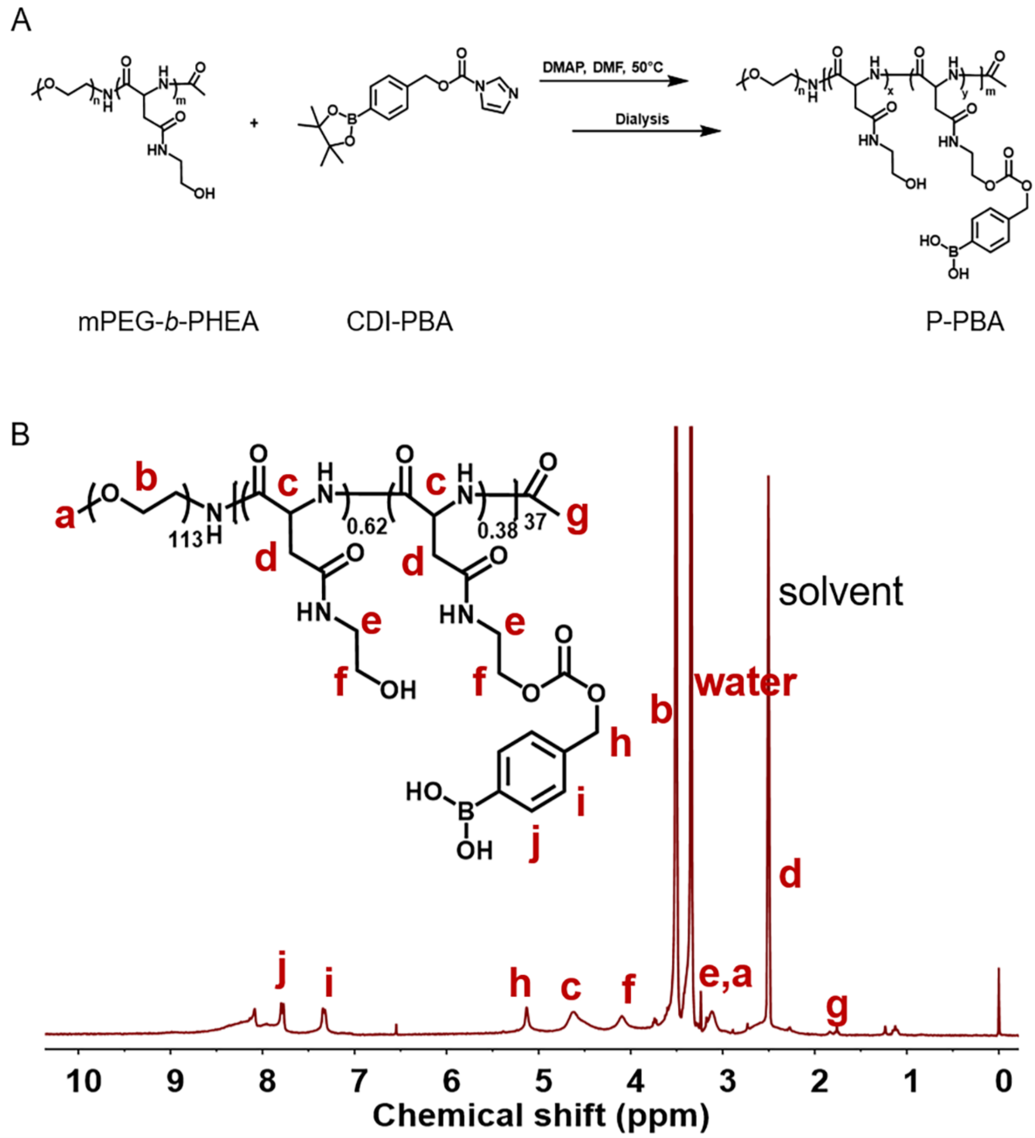

Figure S5 (A)Synthesized route of methoxy poly(ethylene glycol) block poly-[(N-2-hydroxyethyl)-aspartamide] phenylboronic acid (mPEG-*b*-PHEA-PBA, P-PBA). (B) ^{1}H NMR spectrum of methoxy poly(ethylene glycol) block poly-[(N-2-hydroxyethyl)-aspartamide] phenylboronic acid (mPEG-*b*-PHEA-PBA, P-PBA).

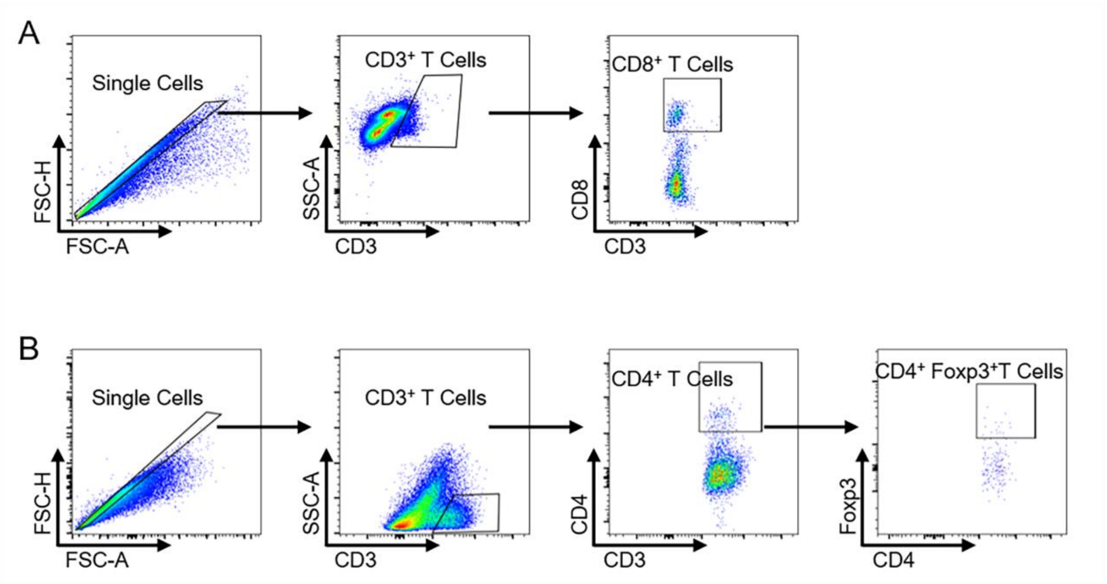

Figure S6 Flow cytometry analysis of the tumor samples after different treatments.

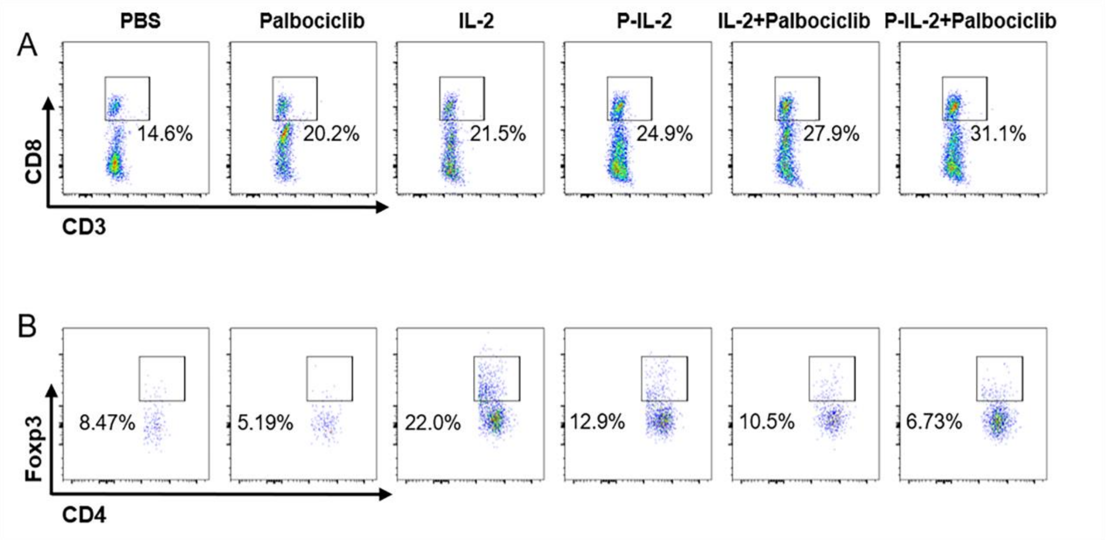

Figure S7 The change of Immune microenvironment in the tumor after different treatments. (A) Intratumoral $CD8^+$ T cells (gated on $CD3^+$ cells), (B) Intratumoral Treg cells (gated on $CD3^+CD4^+$ T cells).

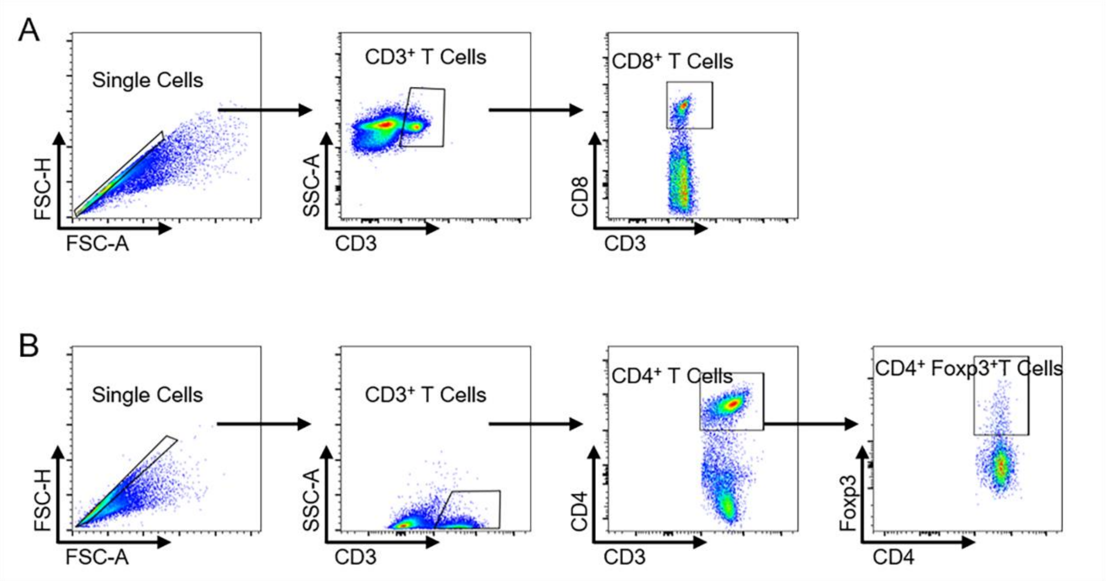

Figure S8 Flow cytometry analysis of the spleen samples after different treatments.

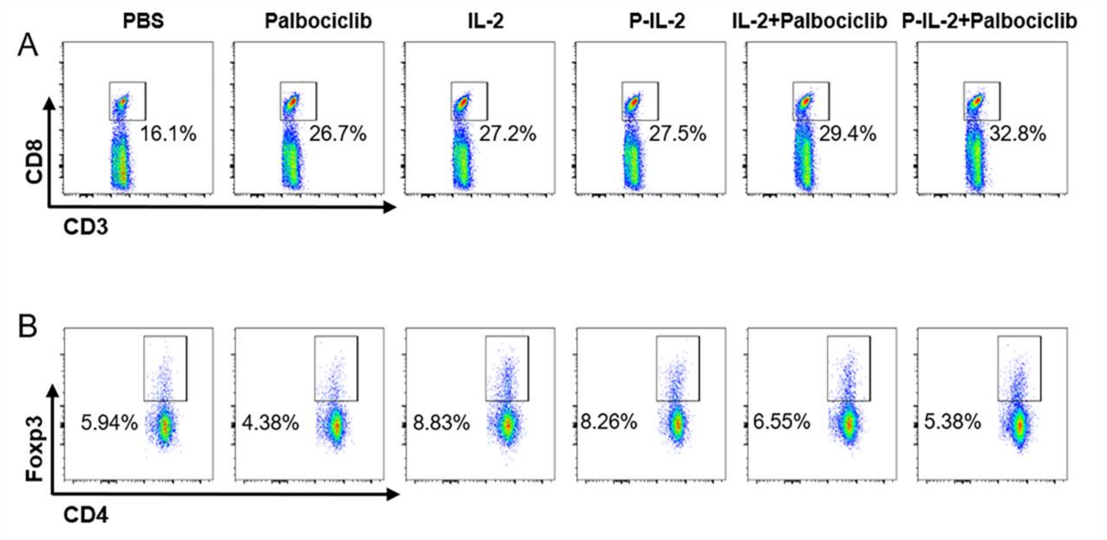

Figure S9 The change of Immune microenvironment in the spleen after different treatments. (A) Splenic $CD8^+$ T cells (gated on $CD3^+$ cells), (B) Splenic Treg cells (gated on $CD3^+CD4^+$ T cells).

Table S1. IL-2 loading efficiency

Groups	Unload IL-2 (%)	DLE (%)	DLC (%)
P-PBA/PLL/IL-2 (32:3:1)	6.4 ± 1.4	93.6 ± 1.4	2.6 ± 0.4
P-PBA/PLL/IL-2 (32:2:1)	6.9 ± 0.4	93.1 ± 0.4	2.6 ± 0.2
P-PBA/PLL/IL-2 (32:1:1)	7.7 ± 0.7	92.3 ± 0.7	2.7 ± 0.3

Table S2. Antibodies used for flow cytometry

Antibodies	Company	Catalog No.	Dilution
FITC anti-mouse CD3 Antibody	Biolegend	100203	1:500
APC/Cy7 anti-mouse CD4 Antibody	Biolegend	100422	1:500
PE/Cy7 anti-mouse CD8a Antibody	Biolegend	100712	1:500
APC anti-mouse CD8a Antibody	Biolegend	100712	1:500
PE anti-mouse FOXP3 Antibody	Thermo Fisher	12-5773-82	1:50
